# A Rare Presentation of a Rare Entity: Wellens Syndrome with Subtle Terminal *T* Wave Changes

**DOI:** 10.1155/2019/1582030

**Published:** 2019-09-16

**Authors:** Mohammed J. Arisha, Ahmad Hallak, Ahmad Khan

**Affiliations:** Department of Internal Medicine, West Virginia University Charleston Division, Charleston Area Medical Center, Charleston, WV, USA

## Abstract

Wellens syndrome is an electrocardiographic (ECG) pattern involving *T* waves in precordial leads that was first described in 1982 among a group of patients presenting with unstable angina suggestive of critical stenosis of the proximal left anterior descending (LAD) coronary. It is crucial for emergency physicians and internists to be able to recognize these patterns, as they occur in the symptom-free periods and represent a pre-infarction state that needs early intervention. Type A, which is characterized by biphasic *T* waves, mainly in V2 and V3, poses a significant challenge to recognize the pattern, and failure to do so can lead to devastating outcome. We report a case of type A Wellens syndrome with subtle *T* wave changes that went unnoticed during the initial assessment and led to start off on a wrong foot.

## 1. Introduction

Wellens syndrome, also known as left anterior descending artery (LAD) T-wave inversion syndrome, is an electrocardiographic pattern that suggests critical stenosis of the proximal part of LAD coronary artery. It was first described in 1982 among a group of patients presenting with unstable angina [1]. Two different types of ECG patterns are described. The first pattern (Type A) is less common, however, more specific, and presents as biphasic *T* waves mainly in precordial leads V2 and V3. Contrary to that, the second pattern (Type B) is more common, less specific, easier to recognize, and is characterized by deep, symmetrically inverted *T* waves in the anterior leads [1, 2]. The ability to recognize both these patterns, especially type A, is extremely important, as the ECG changes can be subtle and easily overlooked, which may lead to fatal outcomes [3]. Our case describes a young patient with no prior risk factors for coronary artery disease (CAD) presenting with a very subtle type A Wellens pattern that went unnoticed during the initial assessment.

## 2. Case Report

**Figure 1:**
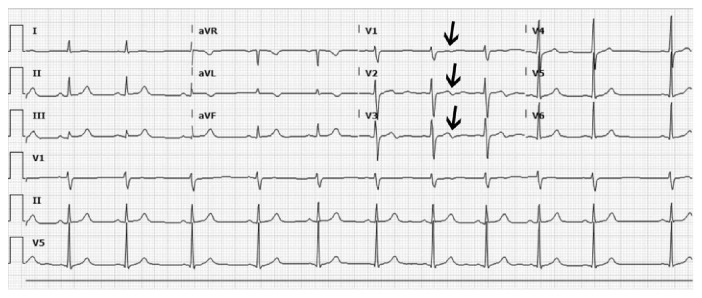
ECG showing subtle biphasic *T* waves in V1–V3.

**Figure 2:**
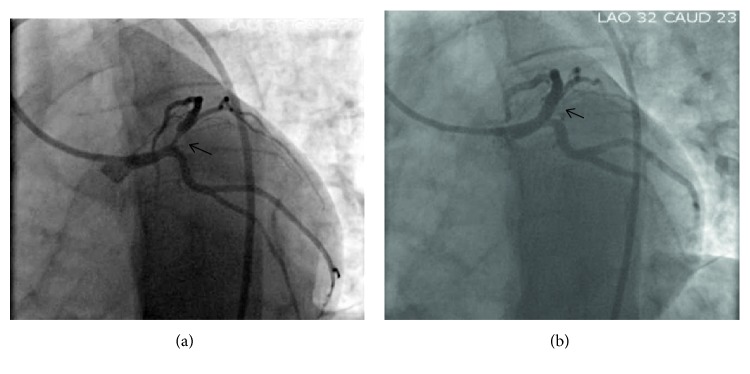
Coronary angiography. Image A shows critical ostial LAD stenosis. Image B shows patent ostial LAD after successful deployment of drug-eluting stent.

A 30-year-old male presented to the Emergency Department (ED) with a one-week history of intermittent atypical substernal chest pain radiating to his jaw with associated palpitations, nausea, and diaphoresis lasting only for few minutes with spontaneous resolution. The patient did not have any significant past medical history. He also didn't have any risk factors for coronary artery disease except for occasional marijuana smoking. His physical examination on presentation was unremarkable. Electrocardiogram (ECG) showed very subtle terminal T-wave inversions in leads V1, V2, and V3 that was read by the computer and cardiologist as “nonspecific T-wave changes” (Figure 1). The first troponin level was 0.05 ng/mL and the subsequent troponin levels were undetectable. The patient was given aspirin 325 mg, atorvastatin, and sublingual nitroglycerin in the ED. He noticed very mild improvement with sublingual nitroglycerin. The patient's HEART score was calculated to be 2, and a decision was made in the ED to initially discharge the patient. However, he developed bradycardia into 40s, and it was decided to admit the patient for overnight monitoring. His repeat ECG did not change much from the previous one. Transthoracic echocardiography was performed and revealed an estimated ejection fraction of 50–55% with no wall motion abnormalities. The admitting team discussed the patient during morning report in the presence of a cardiologist who noticed the subtle abnormality in the patient's ECG. It was decided at that point to consult cardiology who ordered a stress test for the patient. The patient underwent cardiac stress testing, during which he became symptomatic again and developed 5-mm ST-elevation in the anteroseptal leads. Thus, the stress test was stopped prematurely. Subsequently, emergent coronary angiography was performed which showed 95% obstruction in the proximal LAD that was successfully treated with a Drug-Eluting Stent (DES) (Figure 2). The patient was discharged home on optimal medical therapy and remained asymptomatic at nine months.

## 3. Discussion

The interpretation of the ECG is usually the first step in evaluating patients with suspected myocardial ischemia after obtaining a medical history and performing a good physical examination. Thus, it is essential to be able to read and recognize any ECG pattern suggestive of active or impending myocardial ischemia in order to appropriately address and manage the situation in a timely manner. Wellens syndrome is considered a premyocardial infarction state of CAD that is associated with critical LAD stenosis. It was described initially by Wellens and his colleagues in their two original studies published in 1982 and 1989 [1, 2]. Wellens; characteristic ECG patterns were apparent in 26 out of 145 (18%), and in 180 out of 1,260 (14%) patients admitted for unstable angina in the first and second original study, respectively [1, 2]. It was noted that 100% of these patients had LAD lesions, with subsequent development of extensive anterior wall myocardial infarctions among 75% of patients who did not receive coronary revascularization [1]. In a more recent study, the analysis of 424 patients who presented with nonST elevation myocardial infarction (NSTEMI) revealed Wellens ECG patterns in 4.2% of patients. However, only 50% had a culprit LAD lesion [4].

The diagnostic criteria of Wellens syndrome include a history of intermittent chest pain, absent or minimal elevation of cardiac enzymes, and ECG findings of isoelectric or minimally elevated (<1 mm) ST segment, absence of pathological precordial Q waves, biphasic or symmetrically deep inverted *T* waves primarily in leads V2 and V3 [3, 5]. It should be noted that Wellens syndrome has gained more attention recently, and there are several case reports in the English literature describing both ECG patterns. However, types A and B Wellens patterns have been used interchangeably in some reports describing the same ECG changes which reflects some inconsistency [6–9]. They were also labeled as type 1 and 2 in other reports [10, 11]. Thus, we prefer to use the nomenclature mentioned in Wellens original studies that described the less common pattern (type A) as biphasic *T* waves, and the more common pattern (type B) as symmetrically deep inverted *T* waves in the anterior leads, mainly V2 and V3 [1, 2].

Interestingly, Wellens syndrome ECG patterns are apparent during the symptom-free periods, and the early recognition of these patterns is imperative in order to rule out or treat any critical LAD stenosis in a timely manner before extensive anterior myocardial infarction develops. Although not an emergency, as soon as Wellens syndrome is suspected, it is recommended to do an urgent coronary angiography rather than stress test [12]. Provocation of cardiac ischemia in these patients by a stress test can lead to adverse events such as ST elevation [3, 13] as well as fatal outcomes secondary to induction of ventricular tachycardia [3].

Our case highlights the importance of the early recognition of Wellens syndrome which can present with only subtle terminal *T* wave inversions, making it easily overseen. Our patient's ECG changes were subtle enough to go unnoticed by the emergency department physician as well as the reading cardiologist during the initial assessment. Subsequently, it led to inappropriate diagnostic workup with an exercise stress test rather than coronary angiography, which may have resulted in catastrophic outcomes such as serious arrhythmias, acute myocardial infarction, and death. We believe that the recognition of even the most subtle ECG changes along with early intervention may prevent significant morbidity and mortality, and for this reason it is important to remind physicians of this syndrome. Emergency physicians and admitting physicians should advocate for urgent coronary angiography or even coronary CT angiography as opposed to pursuing stress testing in patients with ECG evidence of Wellens Syndrome.

On the other hand, all health care providers should also be aware that these patterns can be recognized in specific clinical scenarios without critical LAD stenosis such as in congenital myocardial bridge, acute cholecystitis, Tako-Tsubo Cardiomyopathy, and substance abuse including cocaine, morphine, phencyclidine, and cannabis, which is called pseudo-Wellens syndrome [14]. Evidence exists to suggest that myocardial edema underlies Wellens ECG pattern. This evidence stems from multiple studies which have correlated Wellens ECG pattern with Cardiac MRI findings in patients with reversible LV systolic dysfunction from nonischemic causes, and both of these findings paralleled in the course of resolution. These findings may suggest the utility of anti-inflammatory agents in the management of patients with nonischemic Wellens Syndrome; however, further studies are necessary to evaluate whether this is a causal relationship [15].

## 4. Conclusion

Terminal *T* wave inversions or biphasic *T* waves in precordial leads represent Type-A Wellens syndrome, which is a rare ECG pattern that suggests high-grade proximal LAD stenosis. These ECG findings can be very subtle and are often interpreted as nonspecific *T* wave changes both by the computer and interpreter, which makes this type of Wellens syndrome easily overseen even by experienced cardiologists. The recognition of these subtle ECG changes is crucial as early intervention may prevent significant morbidity and mortality.
